# Pre‐diagnostic circulating insulin‐like growth factor‐I and bladder cancer risk in the European Prospective Investigation into Cancer and Nutrition

**DOI:** 10.1002/ijc.31650

**Published:** 2018-09-11

**Authors:** Crystal Lin, Ruth C. Travis, Paul N. Appleby, Sarah Tipper, Elisabete Weiderpass, Jenny Chang‐Claude, Inger T. Gram, Rudolf Kaaks, Lambertus A. Kiemeney, Börje Ljungberg, Rosario Tumino, Anne Tjønneland, Nina Roswall, Kim Overvad, Marie‐Christine Boutron‐Ruault, Francesca Romana Manciniveri, Gianluca Severi, Antonia Trichopoulou, Giovanna Masala, Carlotta Sacerdote, Claudia Agnoli, Salvatore Panico, Bas Bueno‐de‐Mesquita, Petra H. Peeters, Elena Salamanca‐Fernández, Maria‐Dolores Chirlaque, Eva Ardanaz, Miren Dorronsoro, Virginia Menéndez, Leila Luján‐Barroso, Fredrik Liedberg, Heinz Freisling, Marc Gunter, Dagfinn Aune, Amanda J. Cross, Elio Riboli, Timothy J. Key, Aurora Perez‐Cornago

**Affiliations:** ^1^ Cancer Epidemiology Unit, Nuffield Department of Population Health University of Oxford Oxford United Kingdom; ^2^ Department of Community Medicine, Faculty of Health Sciences University of Tromsø, The Arctic University of Norway Tromsø Norway; ^3^ Department of Research, Cancer Registry of Norway Institute of Population‐Based Cancer Research Oslo Norway; ^4^ Department of Medical Epidemiology and Biostatistics Karolinska Institutet Stockholm Sweden; ^5^ Genetic Epidemiology Group, Folkhälsan Research Center; Faculty of Medicine University of Helsinki Helsinki Finland; ^6^ German Cancer Research Center (DKFZ) Heidelberg Germany; ^7^ Faculty of Health Sciences, Department of Community Medicine University of Tromsø, The Arctic University of Norway Tromsø Norway; ^8^ Radboud University Medical Center, Department for Health Evidence and Department of Urology Nijmegen The Netherlands; ^9^ Department of Surgical and Perioperative sciences, Urology and Andrology Umeå University Umeå Sweden; ^10^ Cancer Registry and Histopathology Department "Civic ‐ M. P. Arezzo" Hospital Ragusa Italy; ^11^ Danish Cancer Society Research Center Copenhagen Ø Denmark; ^12^ Aarhus University Department of Public Health Section for Epidemiology Aarhus Denmark; ^13^ CESP, Faculté de Médecine UVSQ, INSERM, Université Paris‐Saclay Villejuif France; ^14^ Gustave Roussy Villejuif France; ^15^ Hellenic Health Foundation Athens Greece; ^16^ Cancer Risk Factors and Life‐Style Epidemiology Unit Cancer Research and Prevention Institute, ISPO Florence Italy; ^17^ Unit of Cancer Epidemiology Città della Salute e della Scienza University‐Hospital and Center for Cancer Prevention (CPO) Turin Italy; ^18^ Epidemiology and Prevention Unit Fondazione IRCCS Istituto Nazionale dei Tumori Milan Italy; ^19^ Dipartimento di Medicine Clinica e Chirurgia Federico II University Naples Italy; ^20^ Department for Determinants of Chronic Diseases (DCD) National Institute for Public Health and the Environment (RIVM) Bilthoven The Netherlands; ^21^ Department of Gastroenterology and Hepatology University Medical Centre Utrecht The Netherlands; ^22^ Department of Epidemiology and Biostatistics The School of Public Health, Imperial College London London United Kingdom; ^23^ Department of Social & Preventive Medicine, Faculty of Medicine University of Malaya Kuala Lumpur Malaysia; ^24^ Julius Center for Health Sciences and Primary Care University Medical Center Utrecht Utrecht The Netherlands; ^25^ Escuela Andaluza de Salud Pública, Instituto de Investigación Biosanitaria ibs, GRANADA. Hospitales Universitarios de Granada/Universidad de Granada Granada Spain; ^26^ CIBER Epidemiology and Public Health CIBERESP Madrid Spain; ^27^ Department of Epidemiology, Regional Health Council IMIB‐Arrixaca Murcia Spain; ^28^ Department of Health and Social Sciences Universidad de Murcia Murcia Spain; ^29^ Navarra Public Health Institute Pamplona Spain; ^30^ IdiSNA, Navarra Institute for Health Research Pamplona Spain; ^31^ Public Health Direction and Biodonostia Research Institute‐Ciebersp Basque Regional Health Department Vitoria‐Gasteiz Spain; ^32^ Public Health Directorate Asturias Spain; ^33^ Unit of Nutrition and Cancer, Cancer Epidemiology Research Program Catalan Institute of Oncology‐IDIBELL, L'Hospitalet de Llobregat Barcelona Spain; ^34^ Department of Nursing of Public Health, Mental Health and Maternity and Child Health School of Nursing. Universitat de Barcelona, L'Hospitalet de Llobregat Barcelona Spain; ^35^ Department of Translational Medicine, Lund University and Department of Urology Skåne University Hospital Malmö Sweden; ^36^ Section of Nutrition and Metabolism International Agency for Research on Cancer (IARC‐WHO) Lyon France; ^37^ Bjørknes University College Oslo Norway

**Keywords:** bladder cancer, urothelial cell carcinoma, IGF‐I, EPIC cohort, prospective

## Abstract

Previous *in vitro* and case–control studies have found an association between the insulin‐like growth factor (IGF)‐axis and bladder cancer risk. Circulating concentrations of IGF‐I have also been found to be associated with an increased risk of several cancer types; however, the relationship between pre‐diagnostic circulating IGF‐I concentrations and bladder cancer has never been studied prospectively. We investigated the association of pre‐diagnostic plasma concentrations of IGF‐I with risk of overall bladder cancer and urothelial cell carcinoma (UCC) in a case–control study nested within the European Prospective Investigation into Cancer and Nutrition (EPIC) cohort. A total of 843 men and women diagnosed with bladder cancer between 1992 and 2005 were matched with 843 controls by recruitment centre, sex, age at recruitment, date of blood collection, duration of follow‐up, time of day and fasting status at blood collection using an incidence density sampling protocol. Odds ratios (ORs) and 95% confidence intervals (CIs) were calculated using conditional logistic regression with adjustment for smoking status. No association was found between pre‐diagnostic circulating IGF‐I concentration and overall bladder cancer risk (adjusted OR for highest versus lowest fourth: 0.91, 95% CI: 0.66–1.24, *p*_trend_ = 0.40) or UCC (*n* of cases = 776; 0.91, 0.65–1.26, *p*_trend_ = 0.40). There was no significant evidence of heterogeneity in the association of IGF‐I with bladder cancer risk by tumour aggressiveness, sex, smoking status, or by time between blood collection and diagnosis (*p*_heterogeneity_ > 0.05 for all). This first prospective study indicates no evidence of an association between plasma IGF‐I concentrations and bladder cancer risk.

## Abstract 2

**What's new?**

Past prospective studies have shown a positive association between circulating insulin‐like growth factor I (IGF‐I) concentration and colorectal, prostate, and breast cancer risk. However, the association between circulating IGF‐I concentrations and bladder cancer risk remains uncertain. Using a nested‐case control study with 843 bladder cancer cases across 9 European countries, for the first time here the authors examined prospectively the association between pre‐diagnostic circulating IGF‐I concentrations and bladder cancer risk. IGF‐I was not associated with overall risk of bladder cancer or urothelial cell carcinoma. Further prospective data, including on tumour aggressiveness, are required to examine the association in greater detail.

**Abbreviations**
BMI: body mass index
EPIC: European Prospective Investigation into Cancer and Nutrition
IARC: International Agency for Research on Cancer
IGF‐I: insulin‐like growth factor I
IGF‐IR: insulin‐like growth factor I receptor
LRT: likelihood ratio test
OR: odds ratio
UCC: urothelial cell carcinoma
UK: United Kingdom
VEG‐F: vascular endothelial growth factor

## Introduction

Bladder cancer is the ninth most common cancer worldwide, with 60% of cases occurring in high‐income countries.^1^ There is strong evidence that older age, male sex, family history of bladder cancer, genetic susceptibility, smoking, arsenic in drinking water, occupational exposures to aromatic amines and schistosomiasis infections (only in low‐income countries) are risk factors for bladder cancer.^2^, ^3^ However, the role of other possible risk factors remains unclear.^4^

Insulin‐like growth factor I (IGF‐I) is a peptide hormone that can induce mitosis, prevent apoptosis, promote angiogenesis through vascular endothelial growth factor (VEG‐F), and increase cell migration.^5^ Autocrine IGF‐I signalling from transformed cancerous cells is common, and is an implied mechanism for uncontrolled cell growth.^6^ A number of prospective studies have shown a consistent positive association between circulating IGF‐I concentration and risk of certain cancers such as colorectal, prostate and female breast^7^, ^8^, ^9^ cancer. Previous *in vitro* studies on human bladder cancer cell lines have found that IGF‐I confers a growth advantage to urothelial bladder cancer cells over normal cells.^10^ IGF‐I's receptor, insulin‐like growth factor I receptor (IGF‐IR), has been found to be overexpressed in human bladder cancer cells,^11^ and to play a role in the motility and invasion of bladder cancer cells.^12^ Evidence from a previous case–control study has also suggested that elevated circulating IGF‐I concentrations may be associated with higher risk of bladder cancer.^13^ However, as far as we are aware, the association between circulating IGF‐I concentrations and risk of bladder cancer has not been studied prospectively.

The aim of this study was to investigate the association between pre‐diagnostic circulating concentrations of IGF‐I and risk of overall bladder cancer and urothelial cell carcinoma (UCC) using a case–control study nested within the European Prospective Investigation into Cancer and Nutrition (EPIC) cohort.

## Materials and Methods

### Study population and design

EPIC is a multicentre prospective cohort study of 519,978 participants (153,457 males and 366,521 females), mostly aged 30–75 years. Briefly, subjects were recruited from 23 centres in 10 European countries (Denmark, France, Greece, Germany, Italy, Netherlands, Norway, Spain, Sweden and United Kingdom [UK]) between 1992 and 2000. The original purpose of the cohort was to study the relationship between dietary intake and biomarkers (including hormones) and cancer risk. The majority of participants were recruited from the general population, and were invited to participate based on geographic and administrative boundaries. All EPIC study participants gave written informed consent at recruitment. Approval for the study was granted by the Internal Review Board of the International Agency for Research on Cancer (IARC, Lyon, France) and from ethics committees at participating institutions.^14^

At recruitment, participants provided detailed information on dietary and non‐dietary factors. Approximately 400,000 participants also gave a blood sample that was split into aliquots of plasma, serum, buffy coat and erythrocytes. The aliquots were stored in liquid nitrogen (−196°C) for future laboratory analysis at IARC, with the exception of Denmark and Sweden, where they were stored locally (at −150°C and −70°C, respectively). A more detailed description of subject recruitment, baseline data collection and standard protocols in the EPIC cohort has been previously reported.^14^

Eligibility criteria for this analysis included: (*i*) an available blood sample, (*ii*) information available on the date of blood collection and (*iii*) no history of cancer other than non‐melanoma skin cancer at recruitment.

### Follow‐up and selection of cases and controls

In most countries, incident bladder cancer cases were identified via record linkage to national and regional cancer registries. In France, Germany and Greece, follow‐up was conducted using a variety of methods, including health insurance records, cancer and pathology registries, self‐reported cancer verified with medical records, and active follow‐up through participants and relatives. Follow‐up for these analyses ended between January 2002 (Germany) and October 2005 (Spain).

Cases were eligible for inclusion if they were diagnosed with bladder cancer (International Classification of Disease‐Oncology, Third Edition, topography code C67) between the date of blood collection and end of follow‐up. UCC was defined by morphology codes 812–813. Bladder cancer diagnoses were further characterised by their stage and grade. Tumours with a stage‐grade combination of Ta and Grade 1–2 were considered non‐aggressive, while tumours that were T1 and higher, carcinoma *in situ* or Grade 3 and higher (including Ta) were considered aggressive. A total of 1,861 cases and controls were eligible for matching, of which 150 did not have IGF‐I measurement and 16 had no date of blood collection. The 9 bladder cancer cases from Norway were excluded from this analysis because they either failed to meet the eligibility criteria, or because no suitable control matches were found. The final sample comprised 843 cases and 843 controls. The distribution of bladder cancer cases by EPIC countries can be found in Supporting Information Table S1.

Each bladder cancer case was matched to one control participant, selected at random among all cohort members alive and without any reported cancer diagnosis (except non‐melanoma skin cancer) at date of diagnosis of the index case. Controls were matched based on recruitment centre, sex, age at recruitment (±3 years), date of blood collection (±3 months), time of day of blood collection (±2 hr) and fasting status at blood collection (<3, 3–6, >6 hr). An incidence density sampling protocol was used, such that controls could later become cases if they developed bladder cancer, and each control participant could be sampled more than once.

### Laboratory assay

Pre‐diagnostic plasma IGF‐I concentrations were measured using the automated IDS‐iSYS immunoassay system (Immunodiagnostic Systems Ltd.) at the Cancer Epidemiology Unit laboratory, University of Oxford, UK. As a quality control, two control samples prepared from commercially available pooled plasma (Seralab) were assayed for every 20 study participant samples. Samples from matched case–control sets were analysed within the same batch and laboratory technicians were blinded to case or control status. The intra‐batch coefficient of variation was 2.4%, the inter‐batch coefficient of variation was 3.9% and the overall coefficient of variation was 4.2% at a mean IGF‐I concentration of 13.8 nmol/L. The lower limit of detection was 1.3 nmol/L, adequate to detect the lowest concentration in all study samples.

### Statistical analysis

Baseline characteristics were summarised by their mean and standard deviation, or geometric mean for IGF‐I concentration. Differences in baseline characteristics between cases and control subjects were tested by paired *t*‐test or Wilcoxon's rank sum test for continuous variables, depending on the normality of the distribution. A chi‐square test was used for categorical variables.

For all analyses, circulating IGF‐I concentrations were log transformed to approximate normality. Conditional logistic regression models were used to calculate the odds ratios (ORs) and 95% confidence intervals (CIs) for risk of incident bladder cancer by fourths of circulating IGF‐I concentration, with the lowest fourth as the reference category. All analyses were conditioned on the previously described matching variables.

In the adjusted model, only smoking status, which included intensity (never; former; current: ≤15 cigarettes/day, occasional or cigar smoker; current: >15 cigarettes/day and unknown), was included as a covariate. The following variables were identified *a priori* from the literature^2^ and tested as potential confounders, but did not contribute significantly to model parameters according to likelihood ratio tests (LRTs), and were therefore excluded from the final model: alcohol consumption, total fluid intake, body mass index (BMI), education, physical activity and diabetes. The linear trend for the association of IGF‐I with bladder cancer risk was derived from regression models using the median concentrations within fourths as a continuous variable. The fully‐adjusted final model was also run with a continuous, standardised version of the log IGF‐I variable to determine the risk of bladder cancer per standard deviation (SD) increase in circulating IGF‐I concentration.

To examine possible differences in disease aetiology, a sensitivity analysis was conducted on UCC only, which accounts for the majority of bladder cancer cases.^1^ We also conducted a further sensitivity analysis restricting the model to participants with known smoking status. Subgroup analyses were conducted on subgroups defined *a priori*: sex (male *vs*. female), smoking status (never *vs*. ever), and time from blood collection to diagnosis (<4 *vs*. ≥4 years). To test for heterogeneity, we used LRTs to compare models with and without the interaction term between IGF‐I and the subgroup variable. For tests of heterogeneity of risk by bladder tumour aggressiveness (non‐aggressive *vs*. aggressive), the control in each matched set was assigned the characteristics of their case and the analysis was conducted as described for the subgroups.

All analyses were conducted using Stata statistical software, version 14.1 (Stata Corporation, College Station, TX). Two‐sided *p‐*values are reported, with *p* < 0.05 considered statistically significant.

## Results

The baseline characteristics of the 843 bladder cancer cases and 843 controls are shown in Table 1. Participants were followed up for an average of 5.1 years. The average age at blood collection for both controls and cases was 58 years. For cases, the average age of first bladder cancer diagnosis was 63.6 years. Circulating IGF‐I concentrations did not differ significantly between cases and controls (*p* = 0.2), while smoking history did (*p* < 0.001).

**Table 1:** Characteristics of 843 bladder cancer cases and 843 controls

	Cases (*n =* 843)	Controls (*n =* 843)	*p‐*value^1^
IGF‐I, nmol/L	14.2 (13.9–14.4)^2^	14.3 (14.0–14.6)^2^	0.22^3^
Sex (male)*, n* (%)	613 (72.7%)	613 (72.7%)	–
Age at blood collection, year	58.5 (7.7)	58.4 (7.7)	–
Smoking status*, n* (%)			<0.001^4^
Never	153 (18.1%)	329 (39.0%)	
Former	303 (35.9%)	287 (34.0%)	
Current (≤15 cigarettes/day, other^5^)	227 (26.9%)	154 (18.3%)	
Current (15+ cigarettes/day)	148 (17.6%)	59 (7.0%)	
Unknown	12 (1.4%)	14 (1.7%)	
Physical activity*, n* (%)			0.83^4^
Inactive	221 (26.2%)	207 (24.6%)	
Moderately inactive	273 (32.4%)	288 (34.2%)	
Moderately active	174 (20.6%)	170 (20.2%)	
Active	164 (19.5%)	170 (20.2%)	
Unknown	11 (1.3%)	8 (0.9%)	
Education*, n* (%)			0.63^4^
<Secondary	584 (69.3%)	570 (67.6%)	
Secondary	94 (11.2%)	87 (10.3%)	
Degree	139 (16.5%)	158 (18.7%)	
Unknown	26 (3.1%)	28 (3.3%)	
Body mass index, kg/m^2^	26.7 (4.0)	26.5 (3.8)	0.19
Total energy intake, kcal/day	2,288 (415)	2,293 (434)	0.82
Alcohol intake, mL/day	19.0 (23.3)	17.1 (21.0)	0.25^3^
Cases only			
Age at diagnosis, year	63.6 (8.1)		–
Time between blood collection and diagnosis, year	5.1 (2.8)		–
Tumour aggressiveness*, n* (%)			
Non‐aggressive	344 (40.8%)		–
Aggressive	392 (46.5%)		–
Unknown	107 (12.7%)		–
Urothelial cell carcinoma*, n* (%)	766 (92.1%)		–

Table summarising the main baseline characteristics of the study participants. All values are means (standard deviation) for continuous variables, or *n* (%) when indicated.

1 All values are two‐sided *p‐*value for paired *t*‐test unless otherwise specified.

2 Geometric mean (95% Confidence Interval).

3 *p‐*value for non‐parametric Wilcoxon rank sum test for non‐normally distributed variables.

4 *p‐*value for chi‐square test of association.

5 Other forms of tobacco such as cigars and occasional smokers.

The ORs for overall bladder cancer risk, UCC only and bladder cancer subdivided by aggressiveness by fourths of log IGF‐I, with and without adjustment for smoking status, are shown in Table 2. No association was found between IGF‐I and overall bladder cancer risk (adjusted OR comparing the highest fourth to the lowest fourth of concentration = 0.91, 95% CI: 0.66–1.24, *p*_trend_ = 0.40). When IGF‐I was analysed as a continuous variable, the association between circulating concentrations of IGF‐I and bladder cancer risk remained non‐significant (OR_1SD_ = 0.97; 95% CI: 0.87–1.08; *p*_trend_ = 0.60).

**Table 2:** Odds ratios for bladder cancer risk by fourths of IGF‐I concentration

	Fourths of IGF‐I					
Model	1 (reference)	2	3	4	*p*_trend_ 1	*p*_het_ 2
All bladder cancer cases						–
Cases/controls*, n*	220/202	221/200	199/223	203/218		
OR (95% CI)	1.00 (ref)	0.99 (0.76–1.30)	0.81 (0.62–1.07)	0.83 (0.62–1.11)	0.10	
Adjusted OR (95% CI)^3^	1.00 (ref)	0.99 (0.75–1.34)	0.88 (0.66–1.19)	0.91 (0.66–1.24)	0.40	
Urothelial cell carcinoma only^4^						
Cases/controls*, n*	199/202	208/200	181/223	188/218		
OR (95% CI)	1.00 (ref)	0.98 (0.74–1.30)	0.81 (0.60–1.08)	0.82 (0.61–1.12)	0.11	
Adjusted OR (95% CI)^3^	1.00 (ref)	0.99 (0.73–1.34)	0.86 (0.63–1.18)	0.91 (0.65–1.26)	0.40	
By tumour aggressiveness						
Non‐aggressive^5^						
Cases/controls*, n*	85/85	85/85	83/83	91/91		
OR (95% CI)	1.00 (ref)	1.05 (0.79–1.40)	0.89 (0.66–1.19)	0.82 (0.60–1.12)	0.11	
Adjusted OR (95% CI)^3^	1.00 (ref)	0.82 (0.51–1.31)	0.66 (0.41–1.06)	0.92 (0.55–1.54)	0.40	
Aggressive^6^						
Cases/controls*, n*	103/103	112/112	96/96	81/81		
OR (95% CI)	1.00 (ref)	1.23 (0.79–1.90)	1.36 (0.86–2.16)	0.86 (0.53–1.40)	0.34	
Adjusted OR (95% CI)^3^	1.00 (ref)	1.24 (0.81–1.89)	1.33 (0.85–2.09)	0.86 (0.54–1.39)	0.62	0.06

Odds ratios and 95% confidence intervals for the risk of bladder cancer by fourths of IGF‐I in unadjusted and fully adjusted models. For all analyses, bladder cancer cases and controls were matched on recruitment centre, sex, age at recruitment (±3 years), date of blood collection (±3 months), time of day at blood collection (±2 hr) and fasting status at blood collection (<3, 3–6, >6 hr).

1 *p‐*trend is for a test of linear trend in ORs, derived from regression models using the median concentrations within fourths of log (IGF‐I) as a continuous variable.

2 *p‐*heterogeneity of the adjusted model, calculated using likelihood ratio test comparing models with and without the interaction term.

3 Adjusted model is adjusted for smoking status (never, former, current: ≤15 cigarettes/day, current: >15 cigarettes/day, unknown) and conditioned on the matching variables (above).

4 Urothelial cell carcinoma, defined as ICD‐Oncology, 3rd edition topography code 67 and morphology codes 812–813.

5 Non‐aggressive tumour defined as Stage Ta *and* Grade 1–2.

6 Aggressive tumour defined as ≥ Stage T1 *or* carcinoma in situ *or* ≥ Grade 3

Abbreviations: IGF‐I, insulin‐like growth factor I; UCC, urothelial cell carcinoma.

The ORs were similar when the analyses were restricted to UCC only (0.91, 0.65–1.26, *p*_trend_ = 0.40) and when analyses were restricted to participants with known smoking status **(**Tables 2 and 3). There was no association with risk for either aggressive or non‐aggressive cancers, and no significant heterogeneity by tumour aggressiveness (*p*_heterogeneity_ = 0.06) (Table 2).

**Table 3:** Odds ratios for bladder cancer by fourths of IGF‐I concentration in subgroup and sensitivity analyses

		Adjusted ORs (95% CI) by fourths of IGF‐I					
Model		1 (reference)	2	3	4	*p*_trend_ 1	*p*_heterogeneity_ 2
By sex							
Men	Cases/controls*, n*	147/135	165/138	157/169	144/171		
	Adjusted OR (95% CI)	1.00 (ref)	1.11 (0.78–1.58)	0.97 (0.68–1.37)	0.84 (0.57–1.22)	0.24	
Women	Cases/controls*, n*	73/67	56/62	42/54	59/47		
	Adjusted OR (95% CI)	1.00 (ref)	0.77 (0.46–1.29)	0.67 (0.37–1.20)	1.24 (0.68–2.28)	0.78	0.10
By smoking status							
Never	Cases/controls*, n*	42/76	34/86	41/89	48/91		
	OR (95% CI)^3^	1.00 (ref)	0.69 (0.39–1.21)	0.92 (0.52–1.66)	1.10 (0.62–1.95)	0.99	
Ever	Cases/controls*, n*	175/120	182/110	152/133	152/121		
	OR (95% CI)^3^	1.00 (ref)	1.10 (0.77–1.56)	0.82 (0.58–1.15)	0.83 (0.57–1.20)	0.08	0.13
By time between blood collection and diagnosis							
<4 years since blood collection	Cases/controls*, n*	73/77	88/80	77/74	78/85		
	Adjusted OR (95% CI)	1.00 (ref)	1.08 (0.67–1.76)	1.09 (0.64–1.85)	0.97 (0.57–1.65)	0.93	
≥4 years since blood collection	Cases/controls*, n*	147/125	133/120	122/149	125/133		
	Adjusted OR (95% CI)	1.00 (ref)	0.96 (0.67–1.39)	0.80 (0.56–1.14)	0.89 (0.60–1.33)	0.35	0.79
Restricted to participants with known smoking status	Cases/controls*, n*	216/195	215/196	189/219	198/208		
	Adjusted OR (95% CI)	1.00 (ref)	0.97 (0.75–1.31)	0.83 (0.61–1.12)	0.93 (0.67–1.28)	0.41	

Adjusted odds ratios for smoking status (never, former, current: ≤15 cigarettes/day, current: >15 cigarettes/day, unknown) and conditioned on recruitment centre, sex, age at recruitment (±3 years), date of blood collection (±3 months), time of day at blood collection (±2 hr) and fasting status at blood collection (<3, 3–6, >6 hr).

1 *p‐*trend is for a test of linear trend in ORs, derived from regression models using the median concentrations within fourths of log (IGF‐I) as a continuous variable.

2 *p‐*heterogeneity of adjusted model calculated using likelihood ratio test comparing models with and without the interaction term.

3 ORs and *p‐*heterogeneity calculated using unadjusted model to avoid collinearity by smoking status.

Abbreviations: IGF‐I, insulin‐like growth factor‐I.

Finally, there was no evidence of heterogeneity in the association of IGF‐I and risk of overall bladder cancer by sex (*p*_heterogeneity_ = 0.10), smoking status (*p*_heterogeneity_ = 0.13) or time between blood collection and diagnosis (*p*_heterogeneity=_ 0.79) (Table 3).

## Discussion

The results from this nested case–control study across nine European countries do not suggest an association between pre‐diagnostic circulating concentrations of IGF‐I and risk for bladder cancer. To the best of our knowledge, this is the first prospective investigation into the association between pre‐diagnostic circulating concentrations of IGF‐I and bladder cancer risk.

Previous evidence on the association between IGF‐I and bladder cancer comes from *in vitro* and small case–control human studies. A case–control study of 154 US patients conducted by Zhao et al. in 2003 found patients in the highest fourth of IGF‐I concentration were at increased risk for bladder cancer.^13^ A smaller case–control study by Shariat et al. including 51 US bladder cancer patients and another case–control conducted by Mahmoud et al. with 51 Egyptian bladder cancer patients found no association between IGF‐I levels and bladder cancer.^15^, ^16^ In case–control studies, circulating IGF‐I levels could reflect tumour metabolism rather than a factor influencing risk of developing the disease, since autocrine signalling from tumour cells could elevate IGF‐I levels.^5^

Bladder cancer is a heterogeneous disease. The majority of cases are of the UCC subtype, followed by the squamous cell carcinoma subtype, with different aetiologies.^2^, ^17^ In our sensitivity analysis on UCC only, the OR estimates remained unchanged from the full model, which is unsurprising given that most cases were UCC. Bladder cancer cases can be further divided into non‐aggressive and aggressive tumours, which have been hypothesised to be two separate diseases with distinct molecular signatures.^18^ We found no association with either aggressive or non‐aggressive cancers and no significant heterogeneity in the association by tumour aggressiveness. While genetic studies have suggested that bladder cancer can be classified into more specific molecular subtypes,^19^ we were not able to examine this due to lack of data on tumour genotype.

The strength of this study was the use of prospectively recorded data, which limited any impact of reverse causality on our results. No heterogeneity was observed by time between blood collection and diagnosis, further reducing the possibility of reverse causality. Moreover, a moderately large sample size allowed us to make reasonably precise estimates of the relationship between circulating IGF‐I concentrations and bladder cancer, while information on tumour subtypes enabled us to explore possible heterogeneity in bladder cancer risk by tumour aggressiveness. Finally, the distribution of circulating IGF‐I concentrations among controls in this study was similar to that observed in previous prospective studies.^20^, ^21^

This study has some limitations. First, the analysis relied on a single measurement of circulating IGF‐I in each participant. However, several studies with repeat samples collected between 1 and 5 years apart have seen a moderately high temporal reproducibility of IGF‐I with correlations of 0.7–0.9.^22^, ^23^, ^24^ Therefore, although our analyses may have been affected by regression dilution bias,^25^ this is unlikely to explain the lack of an association. Second, as we did not have information on occupational exposures for the majority of cases and controls, we could not adjust for exposure to industrial chemicals. Third, there were small numbers of cases in subgroups defined by sex, smoking status and tumour aggressiveness, leading to limited statistical power in these analyses. Finally, we were unable to examine data on other IGFs or IGF‐binding proteins, which may interact with and modify the effect of IGF‐I.

In conclusion, there was no evidence of an association between pre‐diagnostic circulating IGF‐I concentrations and bladder cancer risk in the EPIC cohort. To further elucidate the association between circulating IGF‐I concentrations and bladder cancer risk, more data from both prospective and Mendelian randomisation studies are needed, preferably with data on tumour subtypes and aggressiveness to compare study results and ultimately conduct pooled analysis with a larger sample size.

## Supporting information


**Table S1**. Distribution of participants and bladder cancer cases across nine EPIC countries
