# Supplementary material for: Pre‐diagnostic circulating insulin‐like growth factor‐I and bladder cancer risk in the European Prospective Investigation into Cancer and Nutrition
Source: Int J Cancer. 2018 Sep 11;143(10):2351–8. doi: 10.1002/ijc.31650 (PMC6220964; doi:10.1002/ijc.31650)
Supplement: Supplementary file 1 — Table S1. Distribution of participants and bladder cancer cases across nine EPIC countries [file IJC-143-2351-s001.docx]

| **Table S1.** Distribution of participants and bladder cancer cases across nine EPIC countries | | | | | |
| --- | --- | --- | --- | --- | --- |
| **Country** | **Total** | **Bladder cancer cases** | **UCC only^1^** | **Aggressive cancer^2^** | **Nonaggressive cancer^3^** |
| Denmark | 346 | 173 | 166 | 68 | 91 |
| France | 10 | 5 | 3 | 1 | 3 |
| Germany | 210 | 105 | 93 | 40 | 42 |
| Greece | 50 | 25 | 16 | 9 | 5 |
| Italy | 222 | 111 | 104 | 46 | 49 |
| The Netherlands | 120 | 60 | 58 | 28 | 31 |
| Spain | 194 | 97 | 89 | 67 | 23 |
| Sweden | 300 | 150 | 141 | 72 | 65 |
| United Kingdom | 234 | 117 | 106 | 61 | 35 |
| Total | 1686 | 843^4^ | 776 | 392 | 344 |
| ^1^  Urothelial cell carcinomas, defined by ICD-Oncology, 3^rd^ edition topography code 67 and morphology codes 812-813  ^2^  Aggressive tumours defined as ≥ Stage T1 or carcinoma in situ or ≥ Grade 3  ^3^  Non-aggressive tumours defined as Stage Ta and Grade 1-2  ^4^  Cases missing stage and grade information were unable to be classified by tumour aggressiveness. These cases account for the discrepancy between total bladder cancer cases and the sum of aggressive and non-aggressive cancer cases.  Abbreviations: EPIC: European prospective investigation into cancer and nutrition; UCC: urothelial cell carcinoma | | | | | |
